# Emergence of Extensively Drug-Resistant ST170 Citrobacter portucalensis with Plasmids pK218-KPC, pK218-NDM, and pK218-SHV from a Tertiary Hospital, China

**DOI:** 10.1128/spectrum.02510-22

**Published:** 2022-09-26

**Authors:** Xinhua Luo, Lianhua Yu, Jiao Feng, Jin Zhang, Cheng Zheng, Dakang Hu, Piaopiao Dai, Mengqiao Xu, Piaopiao Li, Ronghai Lin, Kai Mu

**Affiliations:** a Department of Clinical Laboratory Medicine, Taizhou Municipal Hospital, Taizhou, China; b Institutes of Biomedical Sciences, Shanxi University, Taiyuan, China; c Department of Critical Care Medicine, Taizhou Municipal Hospital, Taizhou, China; d State Key Laboratory of Pathogen and Biosecurity, Beijing Institute of Microbiology and Epidemiology, Beijing, People’s Republic of China; University of Pittsburgh School of Medicine

**Keywords:** *Citrobacter portucalensis*, *bla*_NDM-1_, *bla*_KPC-2_, mobile genetic elements, multidrug resistance

## Abstract

The objective of this study is to characterize the molecular mechanism of a clinical carbapenem-resistant Citrobacter portucalensis strain K218, which coproduces KPC and NDM carbapenemases. K218 was isolated from a patient's blood sample in a Chinese tertiary hospital. Carbapenemases were detected by the immunocolloidal gold technique. The MIC values were determined by VITEK2. Whole-genome sequencing was performed on K218 and sequence data were analyzed using phylogenetics and extensive genomic comparison. This study reveals that K218 contains a single 5.08 Mb chromosome (51.8% GC content) and four plasmids, pK218-KPC (106 Kb), pK218-NDM (111 Kb), pK218-SHV (191 Kb), and pK218-NR (5 Kb). Twenty-nine types of antibiotic resistance genes were carried on K218, including *bla*_KPC-2_ harbored on pK218-KPC and *bla*_NDM-1_ harbored on pK218-NDM. Detailed comparison of related plasmids of pK218-KPC, pK218-NDM, and pK218-SHV showed that they shared similar conserved backbone regions, respectively. Comprehensive annotation revealed large accessory modules were recombined on the genome of K218. Further analysis speculated that mobile genetic elements bearing abundant resistance genes facilitated the formation of these accessory modules. In conclusion, this study provides an in-depth understanding of the genomic characterization of K218, an extensively drug-resistant *C. portucalensis* strain coproducing NDM and KPC carbapenemase. To the best of our knowledge, this is the first report of *C. portucalensis* strain coharboring *bla*_KPC-2_ and *bla*_NDM-1_ from the clinical setting.

**IMPORTANCE** This is the first report of extensively drug-resistant *C. portucalensis* harboring both *bla*_KPC-2_ and *bla*_NDM-1_. This study will not only extend the understanding of the structural dissection of plasmids and chromosomes carried in *C. portucalensis*, but also expand knowledge of the genetic environment of the *bla*_KPC-2_ and *bla*_NDM-1_ genes. *bla*_KPC-2_ and *bla*_NDM-1_ genes have been suggested to facilitate the propagation and persistence of their host bacteria under different antimicrobial selection pressures. Large accessory regions carrying *bla*_KPC-2_ and *bla*_NDM-1_ genes have become hot spots for transposition and integration, and their structural variation and evolution should receive attention. The multidrug-resistant plasmids pK218-KPC, pK218-NDM, and pK218-SHV with several multidrug resistance regions and the chromosome cK218 with two novel transposons Tn*7410* and Tn*7411* contribute to the formation of extensively drug-resistant *C. portucalensis*.

## INTRODUCTION

Citrobacter portucalensis, recently identified as an emerging species in the *Citrobacter* genus, is a Gram-negative facultative anaerobic bacterium of the *Enterobacteriaceae* family (1). In 2017, strain A60 isolated from aquatic ecosystems in Cantanhede, Portugal, was identified as *C. portucalensis*, a novel species according to phenotype, genotype, and phylogenetic analysis (1). Since then, a series of multidrug-resistant *C. portucalensis* strains have been reported. In 2018, a highly multidrug-resistant *C. portucalensis* strain MBTC-1222 was isolated from vegetables (uziza leaves) in Nigeria and analyzed by whole-genome sequencing, which showed that both A60 and MBTC-1222 carried genes *bla*_CMY_ (encoding β-lactamase that contribute to broad-spectrum β-lactams resistance) and *qnrB*, and MBTC-1222 also carried *bla*_TEM_ (encoding β-lactamase) (2). In 2019, a genome-wide analysis of *C. portucalensis* strain NR-12 of poultry origin in Bangladesh was performed and extensive antimicrobial resistance genes (including *bla*_CMY-39_ and *bla*_TEM-176_) and related mobile genetic elements (MGEs) were identified (3). In 2020, scanning electron micrographs of the *C. portucalensis* strain RIT669 were obtained, and its antibiotic resistance profile was reported (4). In particular, in 2021, a clinically isolated carbapenems-resistant *C. portucalensis* strain 3839 was reported in China. Further analysis showed that strain 3839 carried the resistance gene *bla*_NDM_, and that is the first report of *bla*_NDM_-carrying *C. portucalensis* from the clinical setting (5). Overall, the growing prevalence of multidrug-resistant *C. portucalensis* strains is becoming a potential threat to human society and public health in recent years.

Ambler class A β-lactamase Klebsiella pneumoniae carbapenemase (KPC) and Ambler class B β-lactamase New Delhi metallo-β-lactamase (NDM), the two most common carbapenemases in *Enterobacteriaceae* (6, 7), can hydrolyze nearly all classes of β-lactam antibiotics. Although KPC or NDM carbapenemases have been widely reported broadly, *Enterobacteriaceae* strains that produce both KPC and NDM are rare. Strains coproducing KPC and NDM are a significant threat to public health due to higher-level resistance to carbapenems. Until now, the strains coproducing KPC and NDM have been sporadically reported in *Enterobacteriaceae* such as K. pneumoniae, Escherichia coli, Raoultella ornithinolytica, and Enterobacter hormaechei, but not in *C. portucalensis* (8–12).

In this work, a carbapenem-resistant *C. portucalensis* strain K218 coproducing KPC and NDM carbapenemases from a clinical setting was discovered. The whole genome of K218 including the chromosome of K218, two different IncFII plasmids pK218-KPC and pK218-NDM (IncFII:FIB), IncC plasmid pK218-SHV, and unknown type plasmid pK218-NR were sequenced. All the drug-resistant related MGEs of strain K218 were detailed in genetic dissection. Comprehensive genomic comparisons of plasmids pK218-KPC, pK218-NDM, and pK218-SHV with their closely related plasmids were performed, respectively. In addition, two novel transposons Tn*7410* and Tn*7411* integrating into the chromosome of K218 were identified. Overall, this is the first report of *C. portucalensis* strain co-carrying *bla*_KPC-2_ and *bla*_NDM-1_ in a clinical setting, and the analysis in this study will provide insight into the genomic characterization and the structure of antibiotic resistance genes harbored on *C. portucalensis* strains.

## RESULTS

### Species identification and antimicrobial susceptibility test.

On April 27, 2017, a 56-year-old male was admitted to the neurosurgery department with a diagnosis of left thalamic hemorrhage into the ventricle, hydrocephalus, and hypertension due to lopsided walking with vomiting for 4 h. After admission, craniotomy was performed, blood transfusion, fluid replacement, brain protection, prevention of ulceration and bleeding infection, and neurological rehabilitation were given. On the fifth day postadmission, a carbapenem-resistant *Citrobacter* strain K218 was isolated from the patient's blood sample and initially identified by Vitek 2. Later, bacterial species identification was performed using genome sequence-based average nucleotide identity (ANI) analysis (http://www.ezbiocloud.net/tools/ani), which finally proved that K218 belongs to *C. portucalensis* (13).

The results of antimicrobial susceptibility tests on strain K218 showed that K218 was extensively drug-resistant, exhibiting resistance to all tested antimicrobials except tigecycline and colistin (Table 1). Consistent with the results of the antibiotic susceptibility test, the production of both KPC and NDM carbapenemases was confirmed by the immunocolloidal gold technique (Fig. S1). Corresponding to the multidrug resistance profile results, K218 contained 29 types of resistance genes, all of which are listed in Table S1, conferring resistance to aminoglycosides (*aacA4*cr, *aadA1*, *aphA1*, *armA*, *rmtC*, *strA*, and *strB*), β-lactams (*bla*_CMY-35_, *bla*_KPC-2_, *bla*_NDM-1_, *bla*_OXA-1_, *bla*_SHV-12_, and *bla*_TEM-1_), quinolones (*qnrB9*), sulfonamides (*sul1* and *sul2*), and other antibiotics listed in Table 1.

**TABLE 1:** Antimicrobial drug susceptibility profiles

Antibiotics	MIC (mg/L)/interpretation			
	K218	K218-NDM-EC600^*d*^	K218-KPC-EC600^*d*^	EC600^*c*^
Ceftazidime	≥64/R^*a*^	≥64/R	32/R	0.5/S^*b*^
Piperacilin/tazobactam	≥128/R	≥128/R	≥128/R	≤4/S
Aztreonam	≥64/R	≤1/S	≥64/R	≤1/S
Imipenem	≥16/R	≥16/R	≥16/R	≤0. 25/S
Meropenem	≥16/R	≥16/R	≥16/R	≤0.25/S
Amikacin	≥64/R	≥64/R	≤2/S	≤2/S
Tobramycin	≥16/R	≥16/R	≤1/S	≤1/S
Ciprofloxacin	≥4/R	≤0.25/S	≤0.25/S	≤0.25/S
Levofloxacin	≥8/R	0.5/S	0.5/S	0.5/S
Tigecycline	2/S	≤0.5/S	≤0.5/S	≤0. 5/S
Doxycycline	≥16/R	2/S	1/S	1/S
Minocycline	≥16/R	≤1/S	≤1/S	≤1/S
Colistin	≤0.5/S	≤0.5/S	≤0.5/S	≤0.5/S
Trimethoprim/sulfamethoxazole	≥320/R	≤20/S	≤20/S	≤20/S

a R, resistant.

b S, sensitive.

c EC600 is a rifampicin-resistant E. coli used as a recipient in conjugal transfer experiments.

d K218-NDM-EC600 and K218-KPC-EC600 are transconjugants formed by the transfer of pK218-NDM and pK218-KPC from the wild-type isolate (susceptible to rifampin) into EC600, respectively.

### Phylogenetic analysis of C. portucalensis K218.

**FIG 1:**
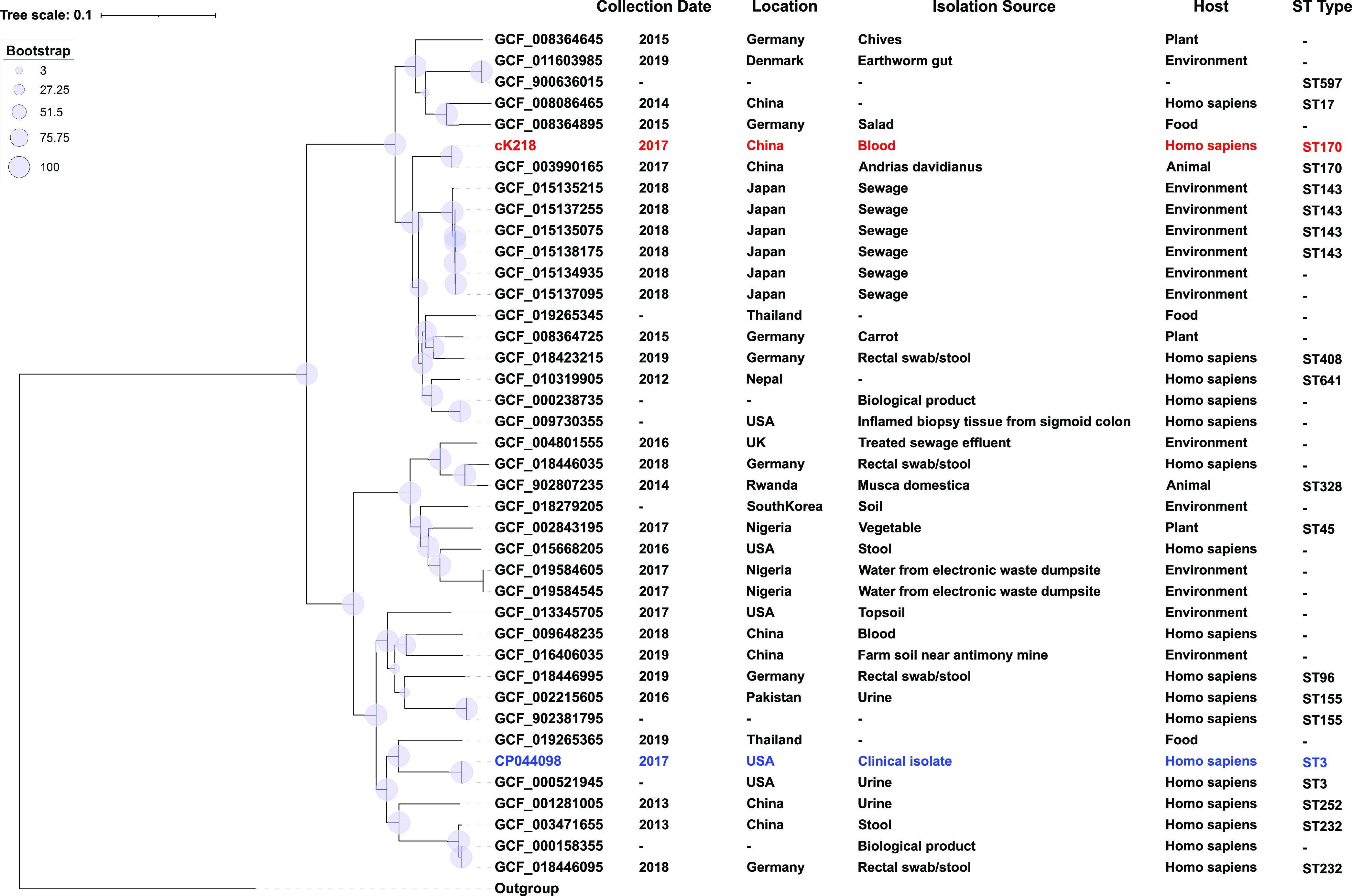
Population distribution of *C. portucalensis* K218 with 39 *C. portucalensis* genomes. The phylogenetic tree was constructed by the Maximum-likelihood method. The degree of support (percentage) for each cluster of associated taxa, as determined by bootstrap analysis, was shown with blue dots next to each branch. The bar corresponded to the scale of sequence divergence. cK218 (the chromosome of K218) was indicated in red and CP044098 was indicated in blue. The annotation denotes (from left to right) were collection date, location, isolation source, host, and ST type. -, not available.

To explore potential associations and the evolutionary relationships between K218 and other *C. portucalensis* strains, phylogenetic analysis was performed based on the core genomes of K218 and all *C. portucalensis* strains from GenBank of NCBI whose assembly levels are scaffold, chromosome, or complete (Table S2) (14). A total of 39 *C. portucalensis* strains from GenBank were included (last accessed on February 16, 2022), and the main collection sites of these strains were China, Germany, the United States, Japan, etc. The chromosome sequence of *C. portucalensis* FDAARGOS_617 (accession number CP044098, the standard strain of *C. portucalensis*) was used as reference. In addition, strain FDAARGOS_549 (accession number CP033744, the standard strain of Citrobacter freundii), was used as the outgroup. In total, 308,026 single nucleotide polymorphisms (SNPs) were identified from these genome sequences. A maximum likelihood (ML) phylogenetic tree was constructed using these SNPs’ data set (Fig. 1). Basic background information such as the collection date, location, isolation source, host, and sequence type (ST) of these strains were marked in Fig. 1. K218 and the standard strain of *C. portucalensis* CP044098 belonged to two main groups, respectively, suggesting that K218 had a less relevant evolutionary relationship with the standard strain CP044098. K218 was most closely related to GCF_003990165, a *C. portucalensis* strain isolated from an *Andrias davidianus* (Chinese giant salamander) in Chongqing China in 2017. They both belonged to the same branch on the evolutionary tree and both of their ST types were ST170. Compared with the average pairwise SNP distance among all strains (50,860 SNPs), these two genomes have a relatively closer relationship with a pairwise SNP distance of 272 SNPs.

### Overview of the C. portucalensis K218.

Genome analysis revealed that K218 contained a chromosome of 5,082,363 bp in length (accession number CP089316), with an average GC content of 51.8%, carrying 4,859 open reading frames (ORFs) (Table 2). Seven key genes (*arcA*, *aspC*, *clpX*, *dnaG*, *fadD*, *lysP*, and *mdh*) on the chromosome of K218 were identified and typed using the MLST method and proved that K218 belonged to ST170. Meanwhile, four circular plasmids were carried in K218, designated as pK218-KPC, pK218-NDM, pK218-SHV, and pK218-NR (Table 2; Fig. S2). Macroscopically, both pK218-KPC and pK218-NDM were IncFII plasmids, although pK218-NDM, encoding two different replication proteins RepA and RepFIB, was more precisely assigned to the IncFII:FIB incompatibility group.

**TABLE 2:** Whole-genome information of *C. portucalensis* K218

Sequence	Mean G+C content (%)	Length (bp)	Total no. of ORFs	MLST	Inc type	Accession no.
cK218	51.8%	5,082,363	4,859	ST170	-^*a*^	CP089316
pK218-KPC	52.8%	105,561	111	-	IncFII	OL988823
pK218-NDM	54.8%	110,709	107	-	IncFII:FIB	OL988824
pK218-SHV	50.5%	190,960	219	-	IncC	OL988825
pK218-NR	52.1%	5,400	11	-	Unknown	OL988826

a not available.

### Genetic characterization of pK218-KPC.

Plasmid pK218-KPC carrying *bla*_KPC_ was 105,561 bp in length, its average GC content was 52.8% and it contained a total of 111 predicted ORFs (Table 2). According to the type of replicon *repA*, pK218-KPC belonged to the IncFII family (15). Accurate annotation and genomic dissection revealed that pK218-KPC can be divided into backbone regions and accessory regions (Fig. S2 and 3). Backbone regions consisted of the major IncFII backbone genes or gene loci such as *repA* (replication), *parA* (partition), *umuCD* (maintenance), and one conjugal transfer region. Accessory regions (13.7-kb *bla*_KPC-2_ region and IS*1X2*) were inserted into different sites of the pK218-KPC backbone regions. *bla*_KPC-2_ carrying in the 13.7-kb *bla*_KPC-2_ region was the only resistance gene that existed on pK218-KPC (Table S1), and pK218-KPC did not contain any resistance genes other than *bla*_KPC-2_.

BLAST analysis of pK218-KPC against the GenBank database showed that pK218-KPC was partially homologous to three plasmids (Fig. S3; Table S3): plasmid p112298-KPC (accession number KP987215, from C. freundii), an unnamed plasmid (accession number CP035635, from Enterobacter cloacae), and another unnamed plasmid (accession number CP073009, from *Citrobacter* sp.). The BLAST comparison of these three plasmids with pK218-KPC showed that all these three plasmids had both 80% to 83% coverage and >98% identity. Strain origins distribution of these plasmids also indicated these three plasmids were mainly distributed within the genus *Citrobacter*.

**FIG 2:**
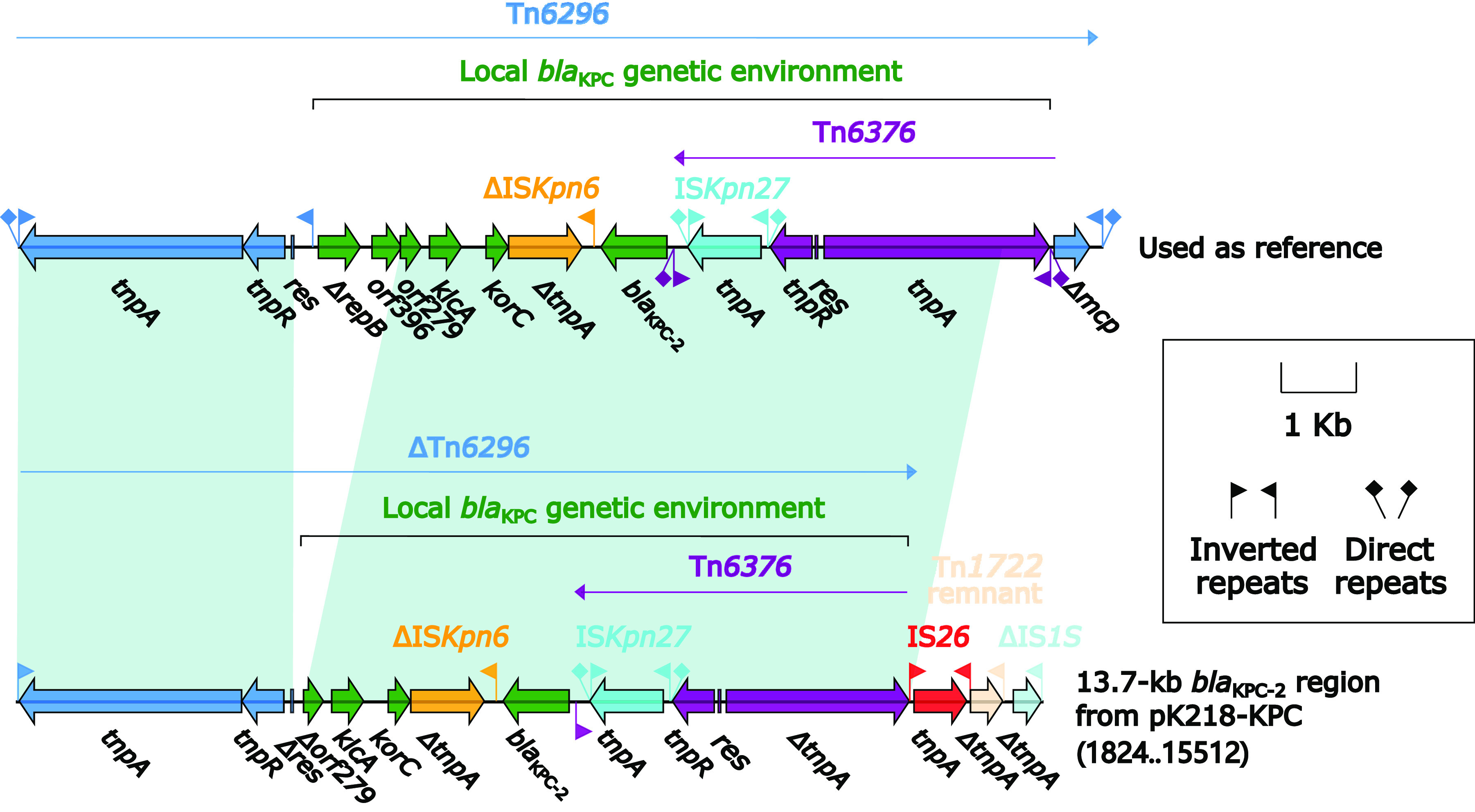
13.7-kb *bla*_KPC-2_ region from pK218-KPC. Genes were denoted by arrows. Genes, mobile genetic elements, and other features were colored based on their functional classification. Shading denoted regions of homology (nucleotide identity > 95%). A single quotation mark in front of the gene name indicated pseudogene. The accession number of Tn*6296* for reference was FJ628167.

Accessory module designated as 13.7-kb blaKPC-2 region was identified in pK218-KPC (Fig. 2). The primary component of the 13.7-kb *bla*_KPC-2_ region was a transposon Tn*6296* with part of genes missing. Tn*6296*, originally identified in plasmid pKP048 from K. pneumoniae, is a unit transposon of the Tn*21* subfamily in the Tn*3* family and is widely considered to be one of the most important vehicles for *bla*_KPC-2_ (16–19). Compared with the intact reference Tn*6296*, a pair of 6-bp direct repeats (DR), target site duplication signals for transposition) at the upstream end were missing in ΔTn*6296*. A 38-bp inverted repeat left (IRL) existed upstream of the interior of ΔTn*6296*. Following the IRL was the core module of the Tn*6296*: *tnpA* (transposase)–*tnpR* (resolvase)–*res* (resolution site). Deletion of a sequence fragment (*orf396–*Δ*repB–IRR*) occurred within ΔTn*6296*, and truncation of part of *tnpA* from Tn*6376* and Δ*mcp* resulted in an interruption of the *tnpA*. The main structure of the local *bla*_KPC_ genetic environment remained Tn*6376*–*bla*_KPC-2_–ΔIS*Kpn6*–*korC*–*klcA–orf279*, and this local environment was consistent with the relevant region of Tn*6296*. The following IS*26*–Tn*1722* remnant–ΔIS*1S* together formed the end of the 13.7-kb *bla*_KPC-2_ region.

### Genetic characterization of pK218-NDM.

Plasmid pK218-NDM carrying *bla*_NDM-1_ was 110,709 bp in length, with an average GC content of 54.8%, and it contained 107 predicted ORFs (Table 2). Although pK218-NDM was an IncFII plasmid, it was more precisely assigned to the IncFII:FIB incompatibility group. The structure of pK218-NDM can also be divided into backbone regions and accessory regions (Fig. S2 and 4), and its backbone regions contained key maintenance genes such as *repA*, *repFIB*, *parA*, *umuCD*, and one conjugal transfer region. However, unlike pK218-KPC, accurate annotation demonstrated that a massive amount of accessory regions (23.1-kb *bla*_NDM-1_ region, ΔIS*5*–IS*Sen4* region, IS*1R*, IS*26*–ΔIS*5*–IS*1R* region, IS*1X2*, IS*Ec33*, IS*903*, and IS*Kox3*) were inserted at different sites in the backbone regions. 23.1-kb *bla*_NDM-1_ region from pK218-NDM contained the drug-resistance gene *sul1*, *rmtC*, *bla*_NDM-1_, and *ble*_MBL_ (Table S1), and the other regions on pK218-NDM did not contain antibiotic-resistance genes.

BLAST analysis revealed that a total of 39 plasmids in the GenBank database were similar to pK218-NDM (>80% coverage and >99% identity) (Fig. S4), and 11 plasmids among these 39 plasmids were almost identical to pK218-NDM (100% coverage and >99% identity). As listed in Table S4, these 11 plasmids were distributed in E. coli, E. cloacae, K. pneumoniae, Serratia marcescens, and other bacteria species, suggesting a widespread of pK218-NDM related plasmids in various species worldwide. At the same time, these 11 plasmids also demonstrated the stability of the *bla*_NDM-1_ genetic environment and the wide distribution of related plasmids.

**FIG 3:**
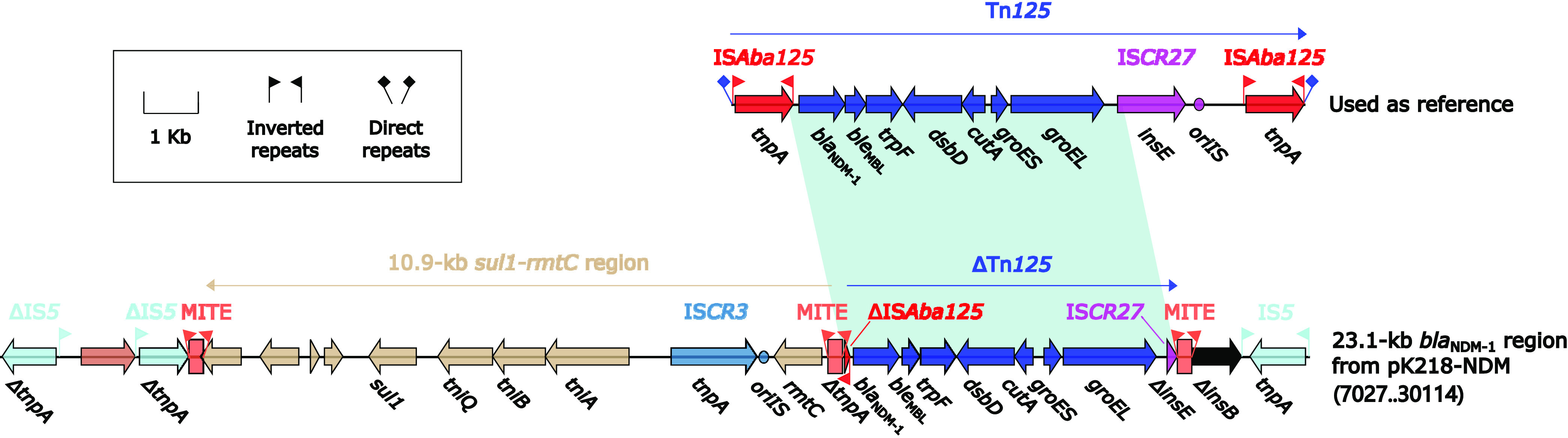
23.1-kb *bla*_NDM-1_ region from pK218-NDM. Genes were denoted by arrows. Genes, mobile genetic elements, and other features were colored based on their functional classification. Shading denoted regions of homology (nucleotide identity > 95%). A single quotation mark in front of the gene name indicated pseudogene. The accession number of Tn*125* for reference was JN872328.

pK218-NDM contained a complex multidrug-resistance (MDR) region harboring *bla*_NDM-1_ designated as a 23.1 kb *bla*_NDM-1_ region (Fig. 3). The genetic contexts of the 23.1-kb *bla*_NDM-1_ region shared a common structure in the above-mentioned 11 plasmids related to pK218-NDM. The upstream of the 23.1-kb *bla*_NDM-1_ region was composed of ΔIS*5*–unknown gene–ΔIS*5*, following a 10.9-kb *sul1–rmtC* region composed of *sul1*–*tniQ–tniB–tniA*–IS*CR3*–*rmtC*. Of these, *sul1* showed resistance to sulfonamides, and *rmtC* showed aminoglycoside resistance. ΔTn*125* harboring *bla*_NDM-1_ and *ble*_MBL_ was a truncated region of Tn*125* (accession number JN872328), leaving only a small part of the original upstream IS*Aba125* and downstream IS*CR27* after truncation. Both ends of ΔTn*125* were flanked by the same miniature inverted-repeat transposable elements (MITEs) with a length of 256 bp (20, 21), and these two copies of the MITE were reported to form a composite transposon-like element, which can mobilize the intervening genetic contexts (17, 22, 23). In ΔTn*125*, ΔIS*Aba125* was located upstream of *bla*_NDM-1_, while *ble*_MBL_ (a bleomycin resistance gene) was located downstream of *bla*_NDM-1_. A set of several genes *trpF–dsbD–cutA–groES–groEL* and IS*CR27* are located further downstream of *ble*_MBL_. The downstream end of the 23.1-kb *bla*_NDM-1_ region consisted of IS*5*.

### Genetic characterization of pK218-SHV.

pK218-SHV was an IncC plasmid of 190,960 bp in length with an average GC content of 50.5% and 219 predicted ORFs (Table 2). Compared with pK218-KPC and pK218-NDM, the size of the pK218-SHV plasmid was larger. In addition to the basic maintenance genes such as *repA* and *parAB*, the backbone regions of the pK218-SHV also contained three conjugal transfer regions *tra1*/*tra2*/*tra3* (Fig. S2 and 5). There were a total of four accessory regions on pK218-SHV named 34.9-kb MDR-1 region, ΔIS*5708*, 12.9-kb MDR-2 region, and IS*Lead2*. Among them, ΔIS*5708* and IS*Lead2* were two insertion sequences (ISs). 34.9-kb MDR-1 region and 12.9-kb MDR-2 region were two larger accessory regions, both of which contained multiple MGEs and putative resistance units (likely able to mobilize as a whole element), suggesting that they had undergone multiple genetic recombinations. pK218-SHV had the largest number of drug-resistance genes among the plasmids carried in K218. Except for *chrA*, *mph*(A), and *aphA1* located in the 12.9-kb MDR-2 region, drug-resistance genes including *bla*_SHV-12_ and *bla*_OXA-1_ were all located in the 34.9-kb MDR-1 region.

BLAST analysis of pK218-SHV showed that pK218-SHV shared similarity to seven plasmids with 80% to 83% coverage and >99% identity in the GenBank database (Fig. S5; Table S5): pASP-a58 (CP014775, from Aeromonas veronii) (24), pKC3-1/2b (MT560001, from K. pneumoniae), pCf53 (KY887593, from C. freundii), pCf52 (KY887592, from C. freundii), p13ARS_GMH0099 (LR697099, from K. pneumoniae), pKp55 (KY887594, from K. pneumoniae), and pVFN3-blaOXA-193K (CP089604, from Vibrio furnissii). Although no such plasmids with 100% coverage had emerged that were nearly identical to pK218-SHV, plasmids related to pK218-SHV had widely spread and distributed among different bacterial species.

**FIG 4:**
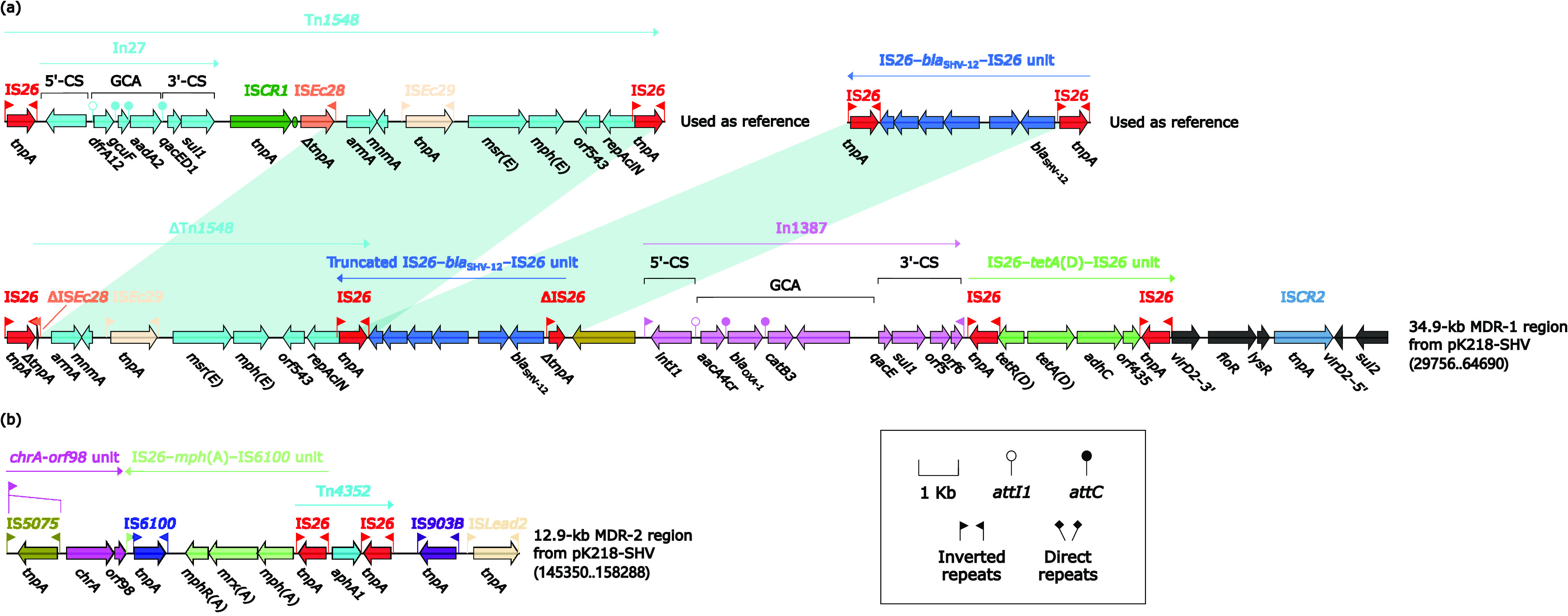
34.9-kb MDR-1 region and 12.9-kb MDR-2 region from pK218-SHV. Genes were denoted by arrows. Genes, mobile genetic elements, and other features were colored based on their functional classification. Shading denoted regions of homology (nucleotide identity > 95%). A single quotation mark in front of the gene name indicated pseudogene. The accession number of Tn*1548* for reference was AF550415.

All drug-resistance genes on pK218-SHV were located in the 34.9-kb MDR-1 region and the 12.9-kb MDR-2 region (Fig. 4). The 34.9-kb MDR-1 region was a complex MDR region composed of multiple MGEs (Fig. 4a). IS*26* was located upstream within the 34.9-kb MDR-1 region followed by ΔTn*1548*. Tn*1548* was a composite transposon first reported in 2005 to be identified as a vector of *armA*, a worldwide disseminated aminoglycoside resistance methylase gene (25). ΔTn*1548* was the remaining part of truncation from the interior of IS*Ec28*, and this truncation also resulted in only a small part of *tnpA* of IS*Ec28* in ΔTn*1548*. Downstream of ΔTn*1548* was the truncated IS*26*–*bla*_SHV-12_–IS*26* unit, which shared the same IS*26* with ΔTn*1548*. The IS*26*–*bla*_SHV-12_–IS*26* unit was likely able to mobilize as a whole module.

After IS*26*–*bla*_SHV-12_–IS*26* unit, In1387, a concise class 1 integron (GCA: *aacA4*cr–*bla*_OXA-1_–*catB3*–gene of unknown designation), was distributed on the 34.9-kb MDR-1 region. The IS*26*–*tetA*(D)–IS*26* unit was distributed after In1387 and was also a whole putative resistance unit although it cannot be considered a composite transposon because DRs were not discovered at both ends. Next, a *floR–lysR*–IS*CR2* fragment was inserted into a *virD2* gene, resulting in the *virD2* being interrupted into 5′ and 3′ ends and reversed. The 34.9-kb MDR-1 region of pK218-SHV was bounded downstream by the *sul2* gene.

The 12.9-kb MDR-2 region consisted of three resistant modules: *chrA–orf98* unit, IS*26–mph*(A)–IS*6100* unit, and Tn*4352* (Fig. 4b). The upstream of Tn*4352* shared the same IS*26* with the IS*26–mph*(A)–IS*6100* unit. The downstream end of the 12.9-kb MDR-2 region was IS*903B* and IS*Lead2*.

### Genetic characterization of pK218-NR.

pK218-NR was the smallest plasmid among all the plasmids of K218; its full length is 5,400 bp, the average GC content was 52.1%, and it contained 11 predicted ORFs (Table 2). Because the replicon gene *rep* was not found on pK218-NR, the Inc type of pK218-NR could not be distinguished. pK218-NR did not contain accessory regions and MGEs, nor did it contain drug-resistance genes.

### Genetic characterization of transposons Tn*7410* and Tn*7411*.

**FIG 5:**
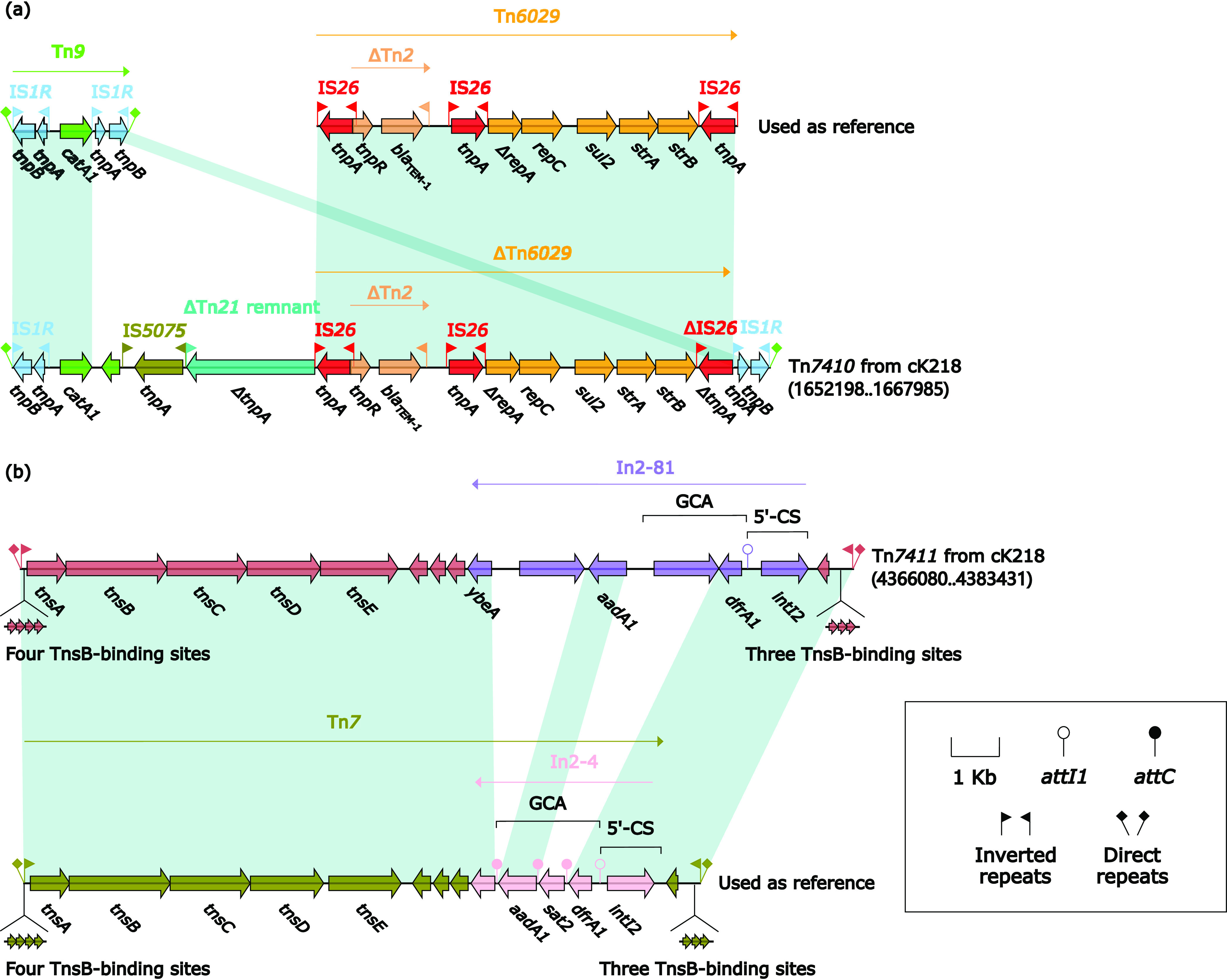
Tn*7410* and Tn*7411* from cK218. Genes were denoted by arrows. Genes, mobile genetic elements, and other features were colored based on their functional classification. Shading denoted regions of homology (nucleotide identity > 95%). A single quotation mark in front of the gene name indicated pseudogene. The accession numbers of Tn*9*, Tn*6029*, and Tn*7* for reference were V00622, GQ150541, and KX117211, respectively. Tn*7* was also present in GCF_003990165 (closely related to cK218 in Fig. 1).

Composite transposon Tn*7410* and unit transposon Tn*7411* were two novel discovered MGEs on chromosome cK218 of K218 (Fig. 5a). Composite transposon Tn*7410* was 15,788 bp in length bounded by two copies of IS*1R*. A pair of 23-bp IRL/IRR and 9-bp DRs flanked Tn*7410*. Due to the structural similarity, Tn*7410* may be formed by the insertion of other MGEs and gene fragments into the IS*1R*-based composite transposon Tn*9* (26). Tn*7410* contained IS*5075*, ΔTn*21* remnant, and ΔTn*6029*. Tn*6029* was an IS*26*-based composite transposon, carrying resistance genes *bla*_TEM-1_, *sul2*, and *strAB* inside (27).

Tn*7411* was 17,352 bp in length and was a unit transposon of the Tn*7* family (Fig. 5b) (28, 29). Tn*7411* had an IRL/IRR of 28 bp at both ends, and a pair of DRs with a length of 5 bp were respectively flanked. There were four repeat regions as TnsB-binding sites upstream of Tn*7411* and three repeat regions as TnsB-binding sites downstream of Tn*7411*. Like other unit transposons from Tn*7* family, Tn*7411* contained the core transposition determents of Tn*7*-family: TnsA (endonuclease), TnsB (transposase), TnsC (transposition regulator), and TnsD plus TnsE (target-site selection proteins) (28, 29). The difference between Tn*7411* and Tn*7* was that In2-4 had been replaced with In2-81. Both In2-4 and In2-81 belonged to class 2 integrons, which were often associated with Tn*7* and its variants. In cassette arrays of In2-81, *sat2* was replaced with gene coding Retron-type RNA-directed DNA polymerase, and no new resistant genes were added. This phenomenon was consistent with previous studies that cassette arrays in class 2 integrons were highly conserved (30). Except for *qnrB9* and *bla*_CMY-35_, all drug-resistance genes on chromosomes were located in Tn*7410* and Tn*7411*.

### Plasmid transfer and resistance phenotypes.

As for conjugation experiments, pK218-KPC and pK218-NDM were transferred from the wild-type isolate (susceptible to rifampin) into E. coli EC600, generating the transconjugant K218-NDM-EC600 and K218-KPC-EC600, respectively. K218-NDM-EC600 was highly resistant to aminoglycosides and carbapenems owing to the presence of *bla*_NDM-1_ and *rmtC* (Table 1). K218-KPC-EC600 was highly resistant to carbapenems due to producing KPC carbapenemase.

## DISCUSSION

Since the first report, as a newly identified bacterial species in recent years, *C. portucalensis* was discovered to be associated with multidrug resistance (1–5), indicating that *C. portucalensis* may naturally be an important repository for drug-resistant genes. Until now, there is only one report on *C. portucalensis* producing NDM in existing reports (5). Thus far, the *C. portucalensis* strain coproducing KPC and NDM carbapenemases have not been reported. To our knowledge, this study is the first report of an extensively drug-resistant *C. portucalensis* strain carrying both *bla*_KPC-2_ and *bla*_NDM-1_. This study would provide an overall in-depth understanding of the genomic characterization of clinically isolated carbapenem-resistant *C. portucalensis* strains.

In previous reports, the hosts of *C. portucalensis* rarely originated from clinical samples, while K218 was obtained from a blood sample of a patient admitted to our hospital. The patient was admitted to the hospital for treatment in April 2017, therefore, making it difficult to identify whether he had contact with animals before admission. From the distribution of the phylogenetic tree (Fig. 1), GCF_003990165 (a *C. portucalensis* strain isolated from *Andrias davidianus*) is most closely related to cK218. Further analysis showed that GCF_003990165 contained only a small number of drug-resistant genes (*aadA1*, *bla*_CMY-35_, *bla*_TEM-1A_, *dfrA1*, *qnrB9*, and *qnrS1*); only contained the IncR plasmid replicon (suggesting that it may only carry the IncR plasmid); its chromosome only carried Tn*7* (Fig. 5), which is the prototype of Tn*7411*, and has the same insertion position as Tn*7411* in K218. These results indicated that wild-derived GCF_003990165 had weak drug resistance and did not have the ability to resist multiple antibiotics. However, K218 had undergone multiple capture, integration, and recombination of MGEs in the hospital. During these events, K218 acquired more abundant drug-resistant genes and stronger drug resistance to face natural selection pressure. Therefore, we speculated that the infection occurred after K218 was transmitted from the animal/environment into the hospital. Furthermore, the patient's medical records showed that K218 was isolated sometime after admission, which supported the conclusion about nosocomial infection.

In this work, the whole-genome sequence of K218 was sequenced and the sequences of its chromosome cK218, IncFII plasmid pK218-KPC, IncFII:FIB plasmid pK218-NDM, IncC plasmid pK218-SHV, unknown type plasmid pK218-NR were obtained, respectively. While both pK218-KPC and pK218-NDM were generalized IncFII plasmids, pK218-NDM belonged to IncFII:FIB in IncFII. Previous research has demonstrated that when FII replicons are associated with FIA or FIB replicons, they can diverge freely because they are not involved in the initiation of plasmid replication (15). pK218-NDM and other IncFII:FIB compatible variants can be used to overcome incompatibility barriers with incoming IncFII plasmids.

For the first three plasmids (pK218-KPC, pK218-NDM, and pK218-SHV), all current corresponding similar plasmids in the GenBank database (with a cut-off value of >80% coverage) were separately included in this study by BLAST analysis (Table S3, 4, and 5). BLAST analysis showed that the identity values of all plasmids included were greater than 98%, indicating that, apart from K218, similar plasmids were also present in other strains and shared conserved backbone regions (Fig. S3, 4, and 5). In particular, for pK218-NDM, a total of 39 plasmids were selected for inclusion in the analysis, of which 11 plasmids had 100% coverage and >99.8% identity. Although these 11 plasmids were carried by different genera of bacteria, they were almost identical to pK218-NDM. The presence of these 11 plasmids illustrated that plasmids related to pK218-NDM plasmids had been widely disseminated and the genetic structure of plasmids related to pK218-NDM was highly stable under different environments. In contrast, for pK218-KPC and pK218-SHV, the maximum coverage of similar plasmids involved was 83%, implying that these two types of plasmids were prone to recombination, deletion, insertion, and transfer of genetic fragments during inheritance and propagation, and their structures were less stable than pK218-NDM. Meanwhile, plasmids related to pK218-KPC were mostly located within the genus *Citrobacter*, whereas plasmids related to pK218-SHV were distributed in several genus, implying that the wide distribution of these three plasmids did not correlate with their structural stability.

Large accessory regions were present on pK218-KPC, pK218-NDM, and pK218-SHV of K218: 13.7-kb *bla*_KPC-2_ region from pK218-KPC, 23.1-kb *bla*_NDM-1_ region from pK218-NDM, 34.9-kb MDR-1 region, and 12.9-kb MDR-2 region from pK218-SHV (Fig. 2, 3, and 4). In these accessory regions, insertion or homologous recombination events mediated by various types of MGEs occurred. At the same time, two novel transposons, Tn*7410* and Tn*7411*, were present on chromosome cK218, and multiple insertions, truncations, and substitutions of MGEs and genetic fragments occurred on these two transposons (Fig. 5). These longer accessory regions have become hot spots for gene truncation and the integration of foreign resistance markers which encoded multiple antibiotic resistance phenotypes. Their structural variation and evolution should receive attention. The multidrug-resistant plasmids pK218-KPC, pK218-NDM, and pK218-SHV with several MDR regions and the chromosome cK218 with two transposons Tn*7410* and Tn*7411* contribute to the formation of extensively drug-resistant K218.

### Conclusion.

In conclusion, we characterized and deciphered the genomic features and population distribution of K218, a newly identified extensively drug-resistant *C. portucalensis* strain harboring both *bla*_KPC-2_ and *bla*_NDM-1_ isolated from the clinical patient. To the best of our knowledge, this is the first report of *C. portucalensis* harboring both *bla*_KPC-2_ and *bla*_NDM-1_. The characterization of K218 will not only significantly extend the understanding of the structural diversification of plasmids and chromosomes carried in K218, but also expand knowledge of the genetic environment of antibiotic resistance genes, especially the *bla*_KPC-2_ and *bla*_NDM-1_ genes. *bla*_KPC-2_ and *bla*_NDM-1_ are drug-resistant genes encoding carbapenemases, and their coexistence will facilitate the propagation and persistence of their host bacteria under different antimicrobial selection pressures. In addition, novel drug-resistant MGEs on chromosomes cannot be ignored during the formation of multidrug-resistant *C. portucalensis*. More whole-genome epidemiological studies are necessary to perform, and active monitoring of extensively drug resistant *C. portucalensis* strains is warranted to prevent these novel strains from further spreading in a hospital environment.

## MATERIALS AND METHODS

### Ethics statement.

The specimens were obtained with the patient’s consent. The use of human specimens and all related experimental protocols were reviewed and approved by the Ethics Committee of Taizhou Municipal Hospital, Zhejiang, China, in accordance with the medical research regulations of the Ministry of Health, China. Research and all related procedures involving biohazardous materials were approved by the Biosafety Committee of Taizhou Municipal Hospital affiliated with Taizhou University. This research was conducted in China.

### Antibiotic susceptibility test and carbapenemases phenotype detection.

The drug MICs of K218, EC600, transconjugant K218-NDM-EC600, and K218-KPC-EC600 were determined by bioMérieux VITEK2 (Table 1). The antibiotic susceptibility test results were determined by the Clinical and Laboratory Standards Institute (CLSI) guidelines (2021).

The production of carbapenemases in K218 was detected on an NG-Test CARBA 5 (Fig. S1). NG-Test CARBA 5 (NG Biotech, Guipry, France), a rapid diagnostic test based on the immunocolloidal gold technique, was used for the detection of the five most common carbapenemase families (KPC, OXA-48-like, VIM, IMP, and NDM).

### Plasmids conjugal transfer.

Conjugal transfer experiments were performed with rifampicin-resistant E. coli EC600 being used as a recipient, and strain K218 as a donor. The donor and recipient strains were grown in 3 mL brain heart infusion (BHI) broth overnight at 37°C. And then, 50 μL of donor strain culture was mixed with 500 μL of recipient strain culture (v:v = 1:10) and 4.5 mL of fresh BHI broth. In addition, 100 μL of the mixture was applied onto a cellulose filter membrane (pore size, 0.22 μm) already placed on a BHI agar plate. After incubation at 37°C for 16 h to 18 h, the filter membrane was taken out and vortexed in 1 mL of BHI broth. The vortex mixtures were plated on BHI agar plates containing 2 mg/L imipenem and 1,500 mg/L rifampicin for the selection of the transconjugants.

### Sequencing and sequence assembly.

The genomic DNA of *C. portucalensis* K218 was extracted using a Gentra Puregene Yeast/Bact. Kit (Qiagen, Valencia, CA). Libraries were prepared separately using the TruePrepTM DNA Library Prep Kit V2 and the SQU-LSK109 Ligation Sequencing kit. After the preparation of the library was completed, it was separately sequenced on an Illumina HiSeq X 10 platform (Illumina Inc., San Diego, CA, USA) and GridION X5 platform (Oxford Nanopore, UK). Raw data from the HiSeq X 10 platform and the GridION X5 platform were trimmed to obtain the high-quality clean reads (clean data) by Canu v1.8 (https://canu.readthedocs.io/en/latest/index.html). The paired-end short Illumina reads and the long Nanopore reads were assembled *de novo* utilizing Unicycler (v0.4.5) (https://github.com/rrwick/Unicycler).

### Whole-genome phylogeny and genetic background analysis.

A total of 39 public sequences of *C. portucalensis* sequenced at the scaffold, chromosome, or complete level were downloaded from NCBI (last accessed on February 16, 2022), which were isolated from various sources from 2012 to 2019. Genomes were aligned against the reference genome to create a core genome alignment using MUMmer v3.1, and a total of 308,026 SNPs in the backbone regions were identified and extracted (31). A maximum-likelihood phylogenetic tree based on the SNPs was constructed. Phylogenetic trees and detailed information (collection date, location, isolation date, host, and ST type) were shown using the Interactive Tree of Life (iTOL) programs (32). The ST types of these *C. portucalensis* genomes were obtained using the web tool PubMLST (https://pubmlst.org).

### Sequence annotation and comparison analysis.

Genome annotation and ORFs/pseudogenes prediction of K218 genomes (cK218, pK218-KPC, pK218-NDM, pK218-SHV, and pK218-NR) were conducted using RAST 2.0 (33). Further manual annotation and detailed dissection were done with BLASTP/BLASTN (34) against the UniProtKB/Swiss-Prot (35) and RefSeq (36). Annotation of drug-resistance genes, MGEs, and other features was performed using online databases such as CARD (37), ResFinder (38), ISfinder (39), and INTEGRALL (40), and the Tn Number Registry (41). Alignments with homologous plasmids sequences of pK218-KPC, pK218-NDM, pK218-SHV, and pK218-NR available in NCBI were performed by using the BRIG tool (42). Genome circle maps and gene organization diagrams of accessory regions were drawn using Inkscape 1.1 (https://inkscape.org/en).

### Data availability.

The data presented in this study are available on request from the corresponding author. The plasmid sequences analyzed in this study can be found in the public NCBI GenBank database. The accession numbers were provided in this article when these plasmids were initially indicated.

The complete sequences of the chromosome of K218 (cK218) and plasmids pK218-KPC, pK218-NDM, pK218-SHV, and pK218-NR were submitted to the GenBank database, under accession numbers CP089316, OL988823, OL988824, OL988825, and OL988826, respectively.
