# Supplementary material for: Emergence of Extensively Drug-Resistant ST170 Citrobacter portucalensis with Plasmids pK218-KPC, pK218-NDM, and pK218-SHV from a Tertiary Hospital, China
Source: Microbiol Spectr. 2022 Sep 26;10(5):e02510-22. doi: 10.1128/spectrum.02510-22 (PMC9603283; doi:10.1128/spectrum.02510-22)
Supplement: Supplemental file 6 — Fig. S1-S5. Download spectrum.02510-22-s0006.pdf, PDF file, 3.0 MB [file spectrum.02510-22-s0006.pdf]

Figure S1. Confirmation of production of both KPC and NDM. The production of carbapenemases in K218 was detected on an NG-Test CARBA 5, a rapid diagnostic test based on the immunocolloidal gold technique.

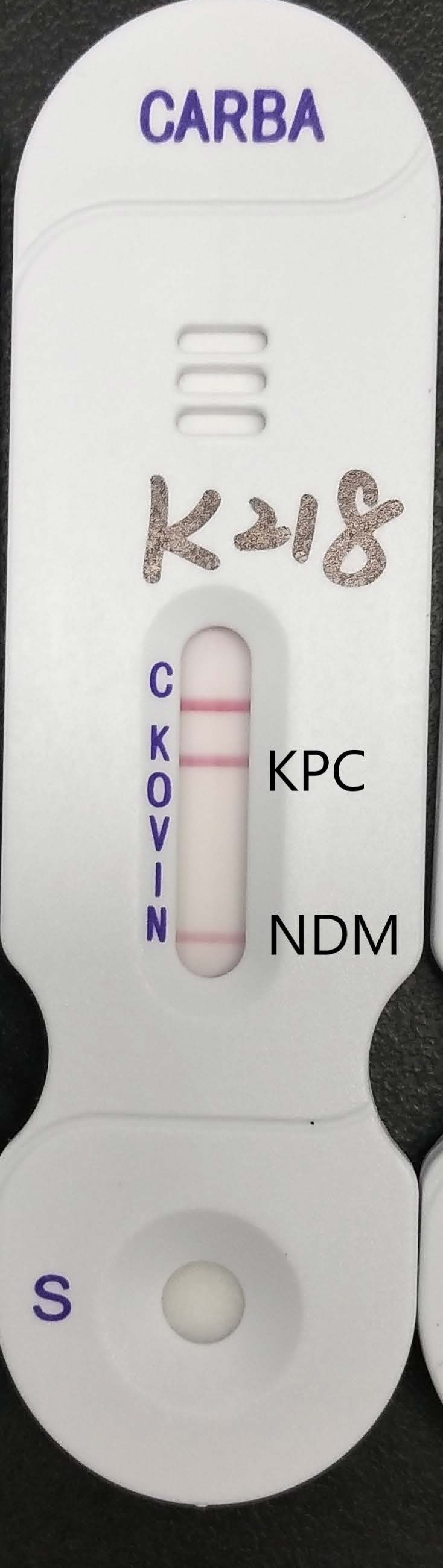
CARBA
K218
C
K
O
V
I
N
KPC
NDM
S

Figure S2. Schematic circle maps of pK218-KPC, pK218-NDM, and pK218-SHV. Genes were denoted by arrows, and the backbone and accessory module regions were highlighted in black and grey, respectively. The innermost circle presented GC-skew [(G-C)/(G+C)], with a window size of 500 bp and a step size of 20 bp. The next-to-innermost circle presented GC content. Backbone regions of conjugal transfer, plasmid maintenance, and plasmid replication were colored in orange, blue, and green, respectively.

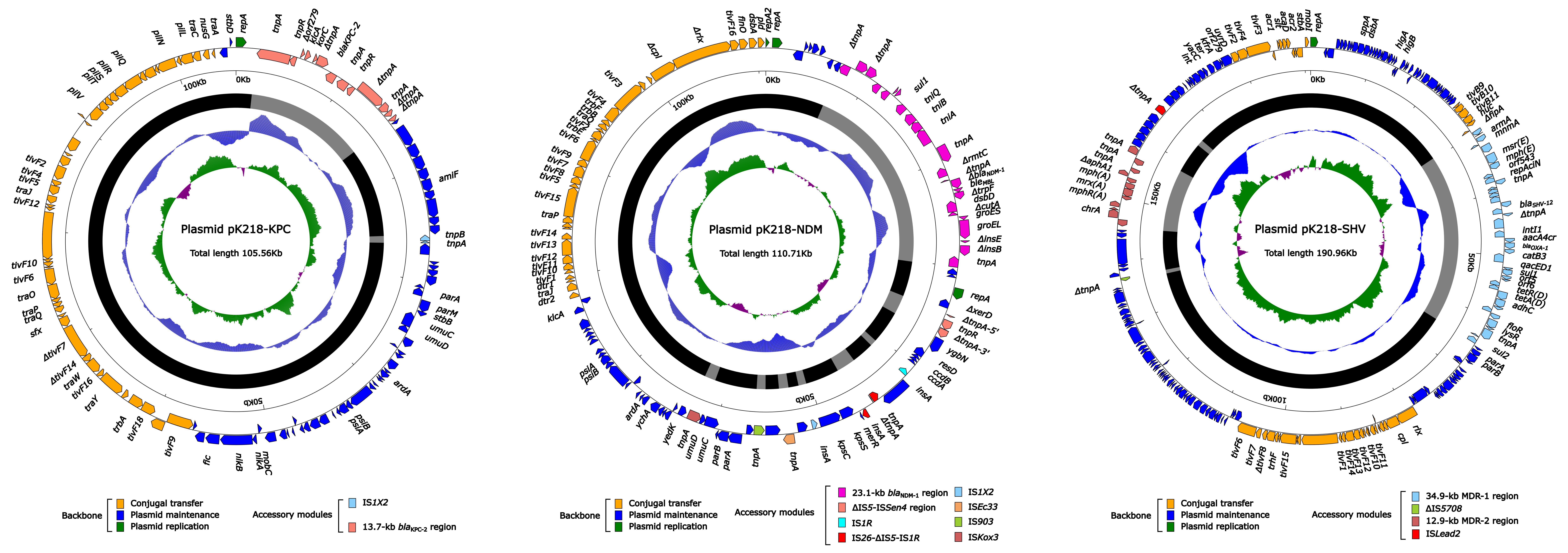

Plasmid pK218-KPC
Total length 105.56Kb
0Kb
50Kb
100Kb
repA
stbD
traA
nusG
traC
pilL
pilN
pilQ
pilR
pilS
pilT
pilV
tnpA
tnpR
Δorf279
klcA
korC
ΔtnpA
blaKPC-2
tnpA
tnpR
ΔtnpA
tnpA
ΔtnpA
ΔtnpA
amiF
tnpB
tnpA
parA
parM
stbB
umuC
umuD
ardA
psiB
psiA
mobC
nikA
nikB
fic
tivF9
tivF18
trbA
traY
tivF16
traW
ΔtivF14
ΔtivF7
sfx
traQ
traP
traO
tivF6
tivF10
tivF12
traJ
tivF5
tivF4
tivF2
Backbone
Conjugal transfer
Plasmid maintenance
Plasmid replication
Accessory modules
IS1X2
13.7-kb blaKPC-2 region
Plasmid pK218-NDM
Total length 110.71Kb
0Kb
50Kb
100Kb
repA
repA2
pld
qsp
finO
tivF16
Δrlx
Δcpl
tivF3
tivF4
tivF6
tivF9
tivF7
tivF8
tivF5
tivF15
traP
tivF14
tivF13
tivF12
tivF11
tivF10
tivF1
dtr1
traJ
dtr2
klcA
psiA
psiB
ardA
ychA
yedK
tnpA
umuD
umuC
parB
parA
tnpA
tnpA
insA
kpsC
kpsS
merR
insA
tnpA
ΔtnpA
insA
ccdA
ccdB
resD
ygbN
ΔtnpA-3'
tnpR
ΔtnpA-5'
ΔxerD
repA
tnpA
ΔinsB
ΔinsE
groEL
groES
ΔcutA
dsbD
ΔtrpF
bleMBL
ΔblaNDM-1
ΔtnpA
ΔrmtC
tnpA
tniA
tniB
tniQ
sul1
ΔtnpA
ΔtnpA
Backbone
Conjugal transfer
Plasmid maintenance
Plasmid replication
Accessory modules
23.1-kb blaNDM-1 region
ΔIS5-ISSen4 region
IS1R
IS26-ΔIS5-IS1R
IS1X2
ISEc33
IS903
ISKox3
Plasmid pK218-SHV
Total length 190.96Kb
0Kb
50Kb
100Kb
150Kb
repA
mobI
stbA
acr2
acpP
slt
acr1
tivF3
tivF4
tivF2
uvrD
orf279
klfA
tetA
yacC
int
ΔtnpA
tnpA
tnpA
tnpA
ΔaphA1
mph(A)
mrx(A)
mphR(A)
chrA
ΔtnpA
spaA
dsbA
higA
higB
tivB9
tivB10
tivB11
fluC
ΔfipA
armA
mnmA
msr(E)
mph(E)
orf543
repAciN
tnpA
blaSHV-12
ΔtnpA
intI1
aacA4cr
blaOXA-1
catB3
qacED1
sul1
orf5
orf6
tetR(D)
tetA(D)
adhC
floR
lysR
tnpA
sul2
parA
parB
rlx
cpl
tivF11
tivF10
tivF12
tivF13
tivF14
tivF1
tivF15
trhF
ΔtivF8
tivF7
tivF6
Backbone
Conjugal transfer
Plasmid maintenance
Plasmid replication
Accessory modules
34.9-kb MDR-1 region
ΔIS5708
12.9-kb MDR-2 region
ISLead2

Figure S3. Alignment of pK218-KPC with related plasmids. pK218-KPC with three other similar plasmids (80-83% coverage and >98% identity) deposited in the GenBank database were included. The rings of plasmids were arranged in the order (from inner to outer) as described in Supplementary Table S3. The third outer ring represented pK218-KPC and the second outer ring colored in gray were annotations of pK218-KPC. Highlighted on the outer ring represented functional regions (backbone regions and accessory regions).

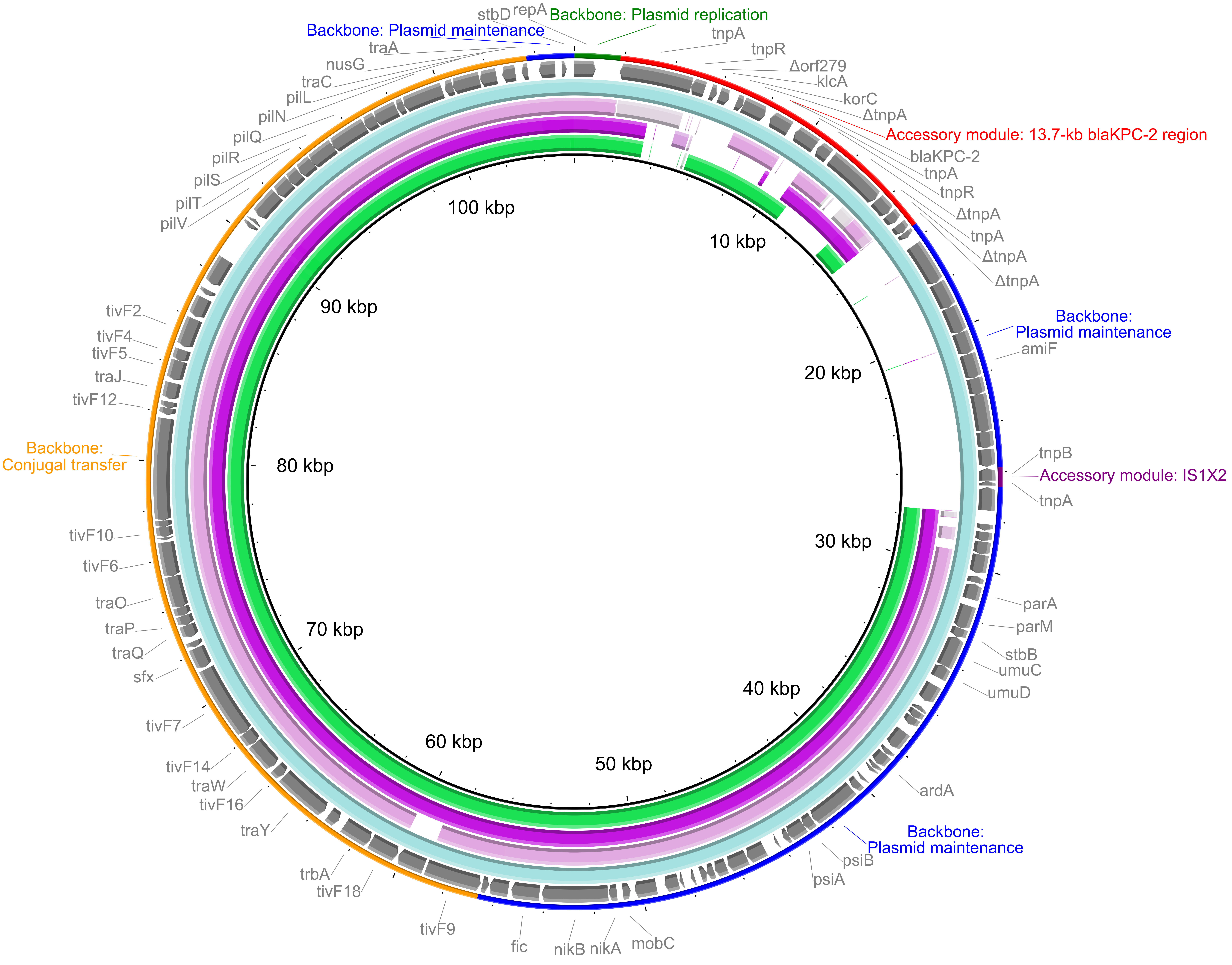

Backbone: Plasmid replication
Backbone: Plasmid maintenance
Accessory module: 13.7-kb blaKPC-2 region
Accessory module: IS1X2
Backbone: Conjugal transfer
stbD
repA
tnpA
tnpR
Δorf279
klcA
korC
ΔtnpA
blaKPC-2
tnpA
tnpR
ΔtnpA
tnpA
ΔtnpA
ΔtnpA
amiF
tnpB
tnpA
parA
parM
stbB
umuC
umuD
ardA
psiB
psiA
mobC
nikA
nikB
fic
tivF9
tivF18
trbA
traY
tivF16
traW
tivF14
tivF7
sfx
traQ
traP
traO
tivF6
tivF10
tivF12
traJ
tivF5
tivF4
tivF2
pilV
pilT
pilS
pilR
pilQ
pilN
pilL
traC
nusG
traA
10 kbp
20 kbp
30 kbp
40 kbp
50 kbp
60 kbp
70 kbp
80 kbp
90 kbp
100 kbp

Figure S4. Alignment of pK218-NDM with related plasmids. pK218-NDM with 39 other similar plasmids (>80% coverage and >99% identity) deposited in the GenBank database were included. The rings of plasmids were arranged in the order (from inner to outer) as described in Supplementary Table S4. The third outer ring represented pK218-NDM and the second outer ring colored in gray were annotations of pK218-NDM. Highlighted on the outer ring represented functional regions (backbone regions and accessory regions).

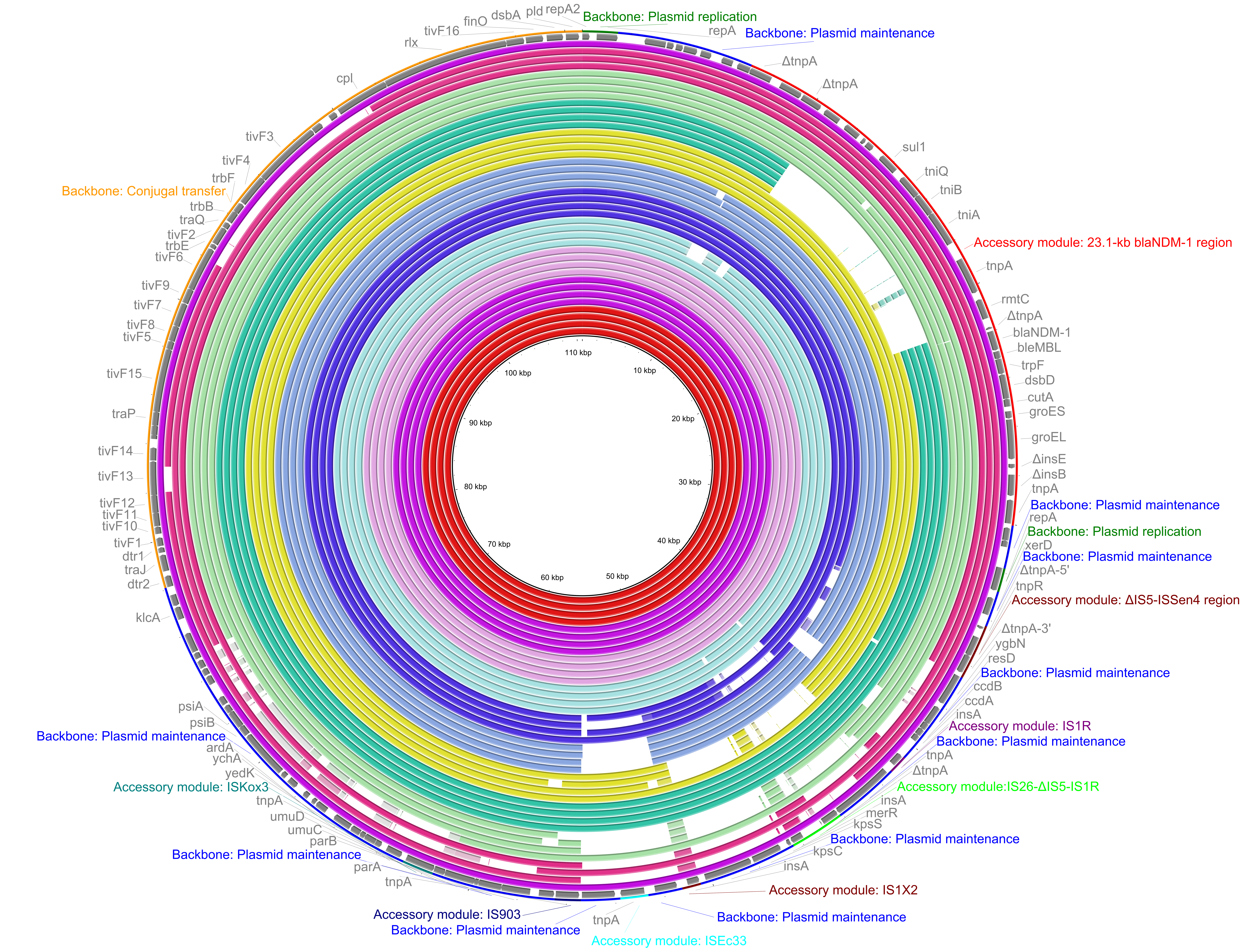
Backbone: Plasmid replication
repA
Backbone: Plasmid maintenance
ΔtnpA
ΔtnpA
sul1
tniQ
tniB
tniA
Accessory module: 23.1-kb blaNDM-1 region
tnpA
rmtC
ΔtnpA
blaNDM-1
bleMBL
trpF
dsbD
cutA
groES
groEL
ΔinsE
ΔinsB
tnpA
Backbone: Plasmid maintenance
repA
Backbone: Plasmid replication
xerD
Backbone: Plasmid maintenance
ΔtnpA-5'
tnpR
Accessory module: ΔIS5-ISSen4 region
ΔtnpA-3'
ygbN
resD
Backbone: Plasmid maintenance
ccdB
ccdA
insA
Accessory module: IS1R
Backbone: Plasmid maintenance
tnpA
ΔtnpA
Accessory module:IS26-ΔIS5-IS1R
insA
merR
kpsS
Backbone: Plasmid maintenance
kpsC
insA
Accessory module: IS1X2
Backbone: Plasmid maintenance
tnpA
Accessory module: ISEc33
Backbone: Plasmid maintenance
Accessory module: IS903
tnpA
parA
Backbone: Plasmid maintenance
parB
umuC
umuD
tnpA
Accessory module: ISKox3
yedK
ychA
ardA
Backbone: Plasmid maintenance
psiB
psiA
klcA
dtr2
traJ
dtr1
tivF1
tivF10
tivF11
tivF12
tivF13
tivF14
traP
tivF15
tivF5
tivF8
tivF7
tivF9
tivF6
trbE
tivF2
traQ
trbB
Backbone: Conjugal transfer
trbF
tivF4
tivF3
cpl
rlx
tivF16
finO
dsbA
pld
repA2
110 kbp
10 kbp
20 kbp
30 kbp
40 kbp
50 kbp
60 kbp
70 kbp
80 kbp
90 kbp
100 kbp

Figure S5. Alignment of pK218-SHV with related plasmids. pK218-SHV with seven other similar plasmids (80-83% coverage and >99% identity) deposited in the GenBank database were included. The rings of plasmids were arranged in the order (from inner to outer) as described in Supplementary Table S5. The third outer ring represented pK218-SHV and the second outer ring colored in gray were annotations of pK218-SHV. Highlighted on the outer ring represented functional regions (backbone regions and accessory regions).

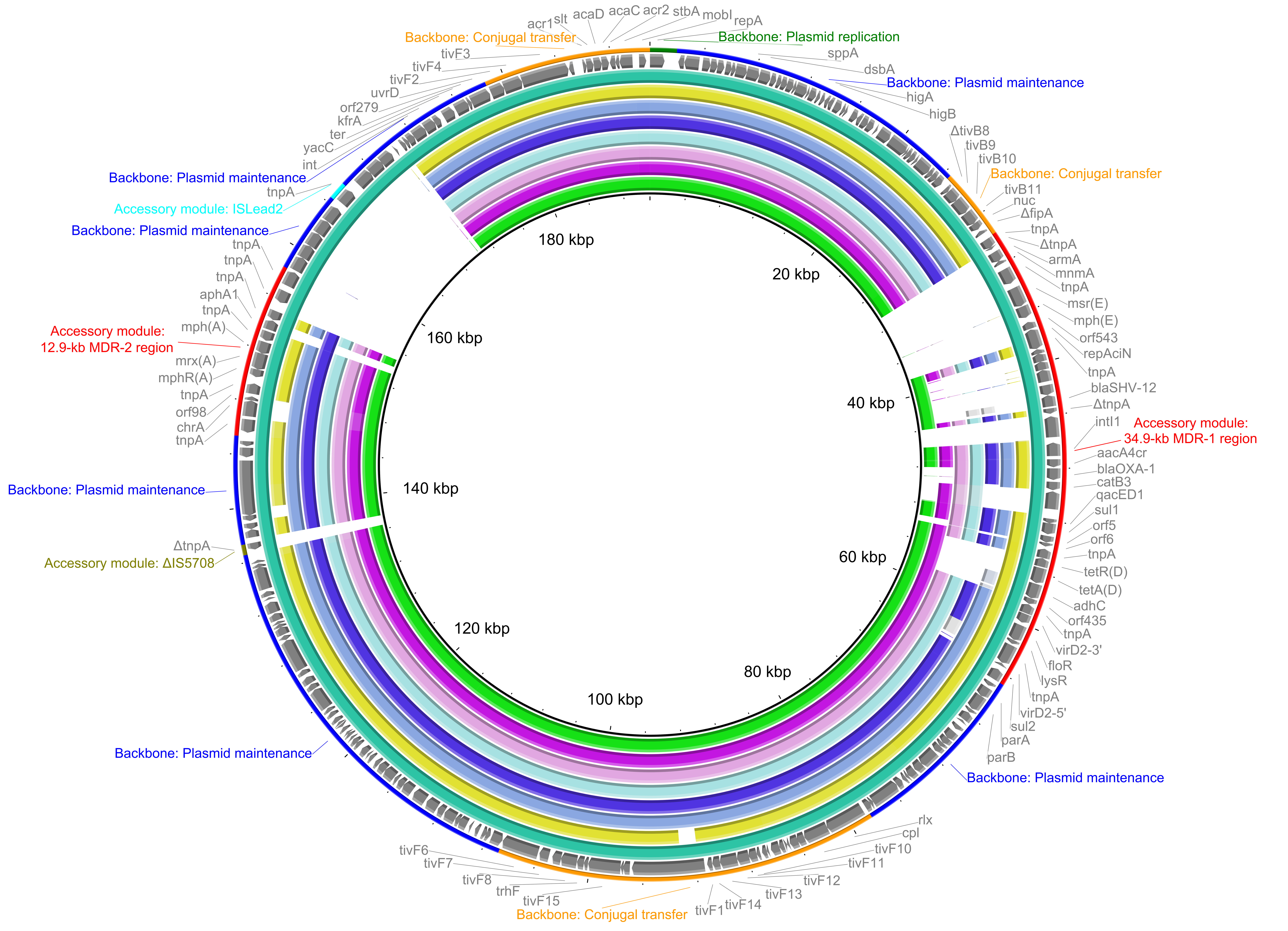

acr1
slt
acaD
acaC
acr2
stbA
mobI
repA
Backbone: Conjugal transfer
Backbone: Plasmid replication
tivF3
sppA
tivF4
dsbA
tivF2
Backbone: Plasmid maintenance
uvrD
higA
orf279
higB
kfrA
ΔtivB8
ter
tivB9
yacC
tivB10
int
Backbone: Conjugal transfer
Backbone: Plasmid maintenance
tivB11
tnpA
nuc
Accessory module: ISLead2
ΔfipA
Backbone: Plasmid maintenance
tnpA
tnpA
ΔtnpA
tnpA
armA
tnpA
mnmA
aphA1
tnpA
tnpA
msr(E)
mph(A)
mph(E)
Accessory module:
12.9-kb MDR-2 region
orf543
repAciN
mrx(A)
tnpA
mphR(A)
blaSHV-12
tnpA
ΔtnpA
orf98
intI1
Accessory module:
34.9-kb MDR-1 region
chrA
tnpA
aacA4cr
blaOXA-1
catB3
qacED1
Backbone: Plasmid maintenance
sul1
orf5
orf6
ΔtnpA
tnpA
Accessory module: ΔIS5708
tetR(D)
tetA(D)
adhC
orf435
tnpA
virD2-3'
floR
lysR
tnpA
virD2-5'
sul2
parA
parB
Backbone: Plasmid maintenance
Backbone: Plasmid maintenance
rlx
cpl
tivF10
tivF11
tivF6
tivF7
tivF12
tivF8
trhF
tivF13
tivF15
tivF14
tivF1
Backbone: Conjugal transfer
20 kbp
40 kbp
60 kbp
80 kbp
100 kbp
120 kbp
140 kbp
160 kbp
180 kbp
